# Structural Dissection of the Maltodextrin Disproportionation Cycle of the *Arabidopsis* Plastidial Disproportionating Enzyme 1 (DPE1)*

**DOI:** 10.1074/jbc.M115.682245

**Published:** 2015-10-26

**Authors:** Ellis C. O'Neill, Clare E. M. Stevenson, Krit Tantanarat, Dimitrios Latousakis, Matthew I. Donaldson, Martin Rejzek, Sergey A. Nepogodiev, Tipaporn Limpaseni, Robert A. Field, David M. Lawson

**Affiliations:** From the ‡ Department of Biological Chemistry, John Innes Centre, Norwich Research Park, Norwich NR4 7UH, United Kingdom and; the § Starch and Cyclodextrin Research Unit, Department of Biochemistry, Faculty of Science, Chulalongkorn University, Bangkok 10330, Thailand

**Keywords:** Arabidopsis, carbohydrate metabolism, chloroplast, crystal structure, glycosyltransferase, acarbose, acarviostatin, cycloamylose, disproportionating enzyme 1, starch degradation

## Abstract

The degradation of transitory starch in the chloroplast to provide fuel for the plant during the night requires a suite of enzymes that generate a series of short chain linear glucans. However, glucans of less than four glucose units are no longer substrates for these enzymes, whereas export from the plastid is only possible in the form of either maltose or glucose. In order to make use of maltotriose, which would otherwise accumulate, disproportionating enzyme 1 (DPE1; a 4-α-glucanotransferase) converts two molecules of maltotriose to a molecule of maltopentaose, which can now be acted on by the degradative enzymes, and one molecule of glucose that can be exported. We have determined the structure of the *Arabidopsis* plastidial DPE1 (AtDPE1), and, through ligand soaking experiments, we have trapped the enzyme in a variety of conformational states. AtDPE1 forms a homodimer with a deep, long, and open-ended active site canyon contained within each subunit. The canyon is divided into donor and acceptor sites with the catalytic residues at their junction; a number of loops around the active site adopt different conformations dependent on the occupancy of these sites. The “gate” is the most dynamic loop and appears to play a role in substrate capture, in particular in the binding of the acceptor molecule. Subtle changes in the configuration of the active site residues may prevent undesirable reactions or abortive hydrolysis of the covalently bound enzyme-substrate intermediate. Together, these observations allow us to delineate the complete AtDPE1 disproportionation cycle in structural terms.

## Teaser

**Background:** *Arabidopsis* maltodextrin disproportionating enzyme 1 (AtDPE1) plays a key role in chloroplast starch degradation.

**Results:** Six AtDPE1 structures define the active site and reveal mechanistically relevant conformations of both the enzyme and substrate.

**Conclusion:** Substrates are captured through loop rearrangements; the subtle deployment of active site residues controls catalysis.

**Significance:** A molecular level understanding of the complete disproportionation cycle of AtDPE1 is presented.

## Introduction

A proportion of the photosynthate generated by plants during the daytime is stored as starch in the chloroplast. At night, this transitory starch is broken down by a suite of degradative enzymes to provide energy for the plant (1–3). Degradation involves the hydrolysis of the α-1,4 and α-1,6 linkages of the starch polymers, principally by β-amylases and debranching enzymes, respectively, releasing short malto-oligosaccharides and glucose, respectively (4–6), although none of these hydrolytic enzymes can act on substrates shorter than four glucose units (*i.e.* maltose and maltotriose) (1, 7). Because only glucose and maltose are exportable to the cytosol, further enzymatic activity is required to prevent the build-up of maltotriose in the chloroplast. This role is fulfilled by disproportionating enzyme 1 (DPE1),^4^ which is the plant plastidial isoform of 4-α-glucanotransferase (EC 2.4.1.25) that is found in all kingdoms of life, although it is referred to as amylomaltase in bacteria. In *Arabidopsis*, the essential role of DPE1 (AtDPE1) was revealed by characterization of a mutant lacking this enzyme; maltotriose accumulated in leaves at night, and the rate of night time starch degradation was reduced (8). Data from a number of plants show that DPE1 enzymes preferentially use maltotriose as a substrate, transferring a maltosyl unit from one molecule to another to generate glucose, which is exportable, and maltopentaose, which can be attacked further by β-amylase or starch phosphorylase (9–13). Additional maltosyl transfer by DPE1 to the newly formed maltopentaose is possible, although more rounds of elongation are less likely *in vivo* because the longer chain glucans become inhibitory to DPE1 activity (10), and these would be efficiently degraded by the aforementioned hydrolytic enzymes.

DPE1 can be used *in vitro* to produce large ring cycloamyloses with degrees of polymerization (DP) in the range of 16–50 (11, 14), although there is no evidence for this occurring *in vivo*. Cycloamyloses have a number of biotechnological applications, including drug (15) or gene (16) delivery, and in the removal of detergents following protein refolding experiments (17). Plant disproportionating enzymes have also been used to transfer glucans from cheap donor molecules onto acceptor molecules (*e.g.* to generate fluorinated oligosaccharides (18) and to generate novel acarviostatin-like molecules (19)). Amylomaltase can also be used to modify starches in order to make thermoreversible gels (20) and alternatives to gelatin and to replace fats in yogurt (21).

DPE1 and amylomaltase belong to the GH77 family, as defined in the CAZy database (22, 23), and, together with GH13, which includes the α-amylases and the cyclodextrin glycosyltransferases, and GH70, which includes the dextransucrases, they form the GH-H clan (24). The structures of several amylomaltases from thermophilic bacteria have been elucidated (25–28), and one unliganded potato enzyme structure has been deposited in the Protein Data Bank (PDB code 1X1N) but is not described in the literature.^5^ Very recently, the structure of the mesophilic amylomaltase from *Escherichia coli* (also referred to as MalQ) has also been reported (29).

Here we present crystal structures of *Arabidopsis thaliana* DPE1 (AtDPE1), revealing both covalently and non-covalently bound complexes of the enzyme with a variety of relevant ligands. As such, this represents the most extensive structural study so far reported for this class of enzyme. We define key protein-ligand interactions and describe a series of conformational changes that are linked to active site occupancy, which together direct the course of catalysis and, crucially, favor transglycosylation over hydrolysis. Moreover, these structures help to explain why maltotriose is the preferred substrate and why longer glucans are inhibitory to catalysis. Overall, our data permit a structural definition of the complete catalytic cycle of the enzyme and allow us to propose a mechanism for the industrially important production of large cyclic products.

## Experimental Procedures

### 

#### 

##### Crystallization and X-ray Data Collection

AtDPE1 protein was prepared as previously described (11) to give a sample corresponding to residues 46–576 of the wild-type amino acid sequence (*i.e.* lacking the N-terminal transit peptide; UniProtKB/Swiss-Prot entry Q9LV91) and preceded by a hexahistidine tag (encoded by the pET151/D-TOPO vector (Invitrogen)). The tag was not cleaved prior to crystallization by the hanging drop vapor diffusion method at 20 °C. 1 μl of a precipitant solution consisting of 9% (w/v) PEG 2000 monomethyl ether in 0.1 m HEPES-NaOH, pH 8.0, was added to 1 μl of protein at a concentration of ∼10 mg/ml in 20 mm HEPES-NaOH, pH 7.5, 150 mm NaCl (protein concentration was estimated on a Nanovue spectrophotometer (GE Healthcare) using an extinction coefficient of 128,590 m^−1^ cm^−1^ calculated from the amino acid composition by ProtParam (30)). Crystals were cryoprotected using the precipitant solution supplemented with 20% (v/v) ethylene glycol in place of an equivalent volume of buffer. Ligand soaking solutions were made up in this cryoprotectant solution at 10 mm for maltotriose (Sigma-Aldrich), acarbose (Toronto Research Chemicals Inc.), and β-cyclodextrin (Wacker-Chemie GmbH); at 0.9 mm for acarviostatin II01 (for production details, see below); and at 1 mg/ml for cycloamylose (Wako Pure Chemical Industries Ltd.). Crystals were soaked overnight in 5-μl drops of these solutions before harvesting and flash-cooling in liquid nitrogen using LithoLoops (Molecular Dimensions). The mounted crystals were stored in Unipuck cassettes (MiTeGen) prior to transport to the Diamond Light Source (Oxfordshire, UK), where they were transferred robotically to the goniostat on either beamline I04 or I04-1 and maintained at −173 °C with a Cryojet cryocooler (Oxford Instruments). X-ray data were recorded using either a Quantum 315 CCD (ADSC) or a Pilatus 2M or 6M (Dectris) detector and then integrated using either MOSFLM (31) or XDS (32) and scaled and merged using SCALA (33) or AIMLESS (34); the resultant data collection statistics are summarized in Table 1.

**TABLE 1:** **X-ray data collection and processing**

Data set	LF	MA3	ACR	ACV	BCD	CAM
Beamline	I04	I04–1	I04–1	I04	I04–1	I04–1
Wavelength (Å)	0.9763	0.9173	0.9173	0.9790	0.9173	0.9173
Detector	ADSC Q315	Pilatus 2M	Pilatus 2M	Pilatus 6M	Pilatus 2M	Pilatus 2M
Resolution range (Å)*^a^*	57.78–2.13 (2.24–2.13)	63.74–1.80 (1.85–1.80)	63.67–2.05 (2.10–2.05)	40.95–2.53 (2.60–2.63)	63.63–2.30 (2.36–2.30)	32.03–2.30 (2.36–2.30)
Space group	P1	P1	P1	P1	P1	P1
*a*, *b*, *c* (Å)	70.65, 74.22, 79.70	69.41, 73.13, 79.12	69.79, 73.69, 79.35	70.83, 74.13, 79.73	69.75, 73.11,79.33	69.89, 73.35, 79.39
α, β, γ (degrees)	64.97, 69.48, 66.02	65.46, 69.48, 67.09	64.59, 69.99, 66.78	64.59, 69.76, 66.57	65.44, 69.48, 66.85	65.28, 69.49, 66.86
Total observations*^a^*	181,754 (23,984)	447,034 (29,906)	303,777 (20,932)	123,306 (8992)	276,058 (20,600)	279,175 (20,509)
Unique reflections*^a^*	68,185 (9066)	105,329 (6956)	72,219 (5162)	42,064 (3104)	52,530 (3901)	52,739 (3900)
Multiplicity*^a^*	2.7 (2.6)	4.2 (4.3)	4.2 (4.1)	2.9 (2.9)	5.3 (5.3)	5.3 (5.3)
Mean *I*/σ(*I*)*^a^*	6.7 (1.7)	20.5 (3.0)	14.3 (1.6)	5.7 (1.9)	11.8 (2.5)	14.3 (3.0)
Completeness (%)*^a^*	92.6 (84.4)	89.5 (79.9)	89.7 (87.0)	95.9 (96.4)	92.5 (92.5)	92.4 (92.2)
*R*_merge_*^a,b^*	0.084 (0.541)	0.036 (0.436)	0.067 (0.847)	0.110 (0.447)	0.081 (0.691)	0.077 (0.552)
*R*_meas_*^a,c^*	0.106 (0.679)	0.041 (0.497)	0.092 (1.019)	0.156 (0.633)	0.102 (0.869)	0.085 (0.613)
*CC*½*^a,d^*	0.992 (0.759)	0.999 (0.884)	0.997 (0.636)	0.984 (0.701)	0.998 (0.783)	0.998 (0.870)
Wilson *B* value (Å^2^)	35.7	27.2	29.7	31.5	41.1	41.0

*^a^* Values for the outer resolution shell are given in parentheses.

*^b^ R*_merge_ = Σ*_hkl_* Σ*_i_* |*I_i_*(*hkl*) − 〈*I*(*hkl*)〉|/Σ*_hkl_* Σ*_i_I_i_*(*hkl*).

*^c^ R*_meas_ = Σ*_hkl_* (*N*/(*N* − 1))½ × Σ*_i_* |*I_i_*(*hkl*) − 〈*I*(*hkl*)〉|/Σ*_hkl_* Σ*_i_I_i_*(*hkl*), where *I_i_*(*hkl*) is the *i*th observation of reflection *hkl*, 〈*I*(*hkl*)〉 is the weighted average intensity for all observations *i* of reflection *hkl*, and *N* is the number of observations of reflection *hkl*.

*^d^ CC*½ is the correlation coefficient between symmetry-related intensities taken from random halves of the data set.

##### Structure Solution and Refinement

**FIGURE 1.**
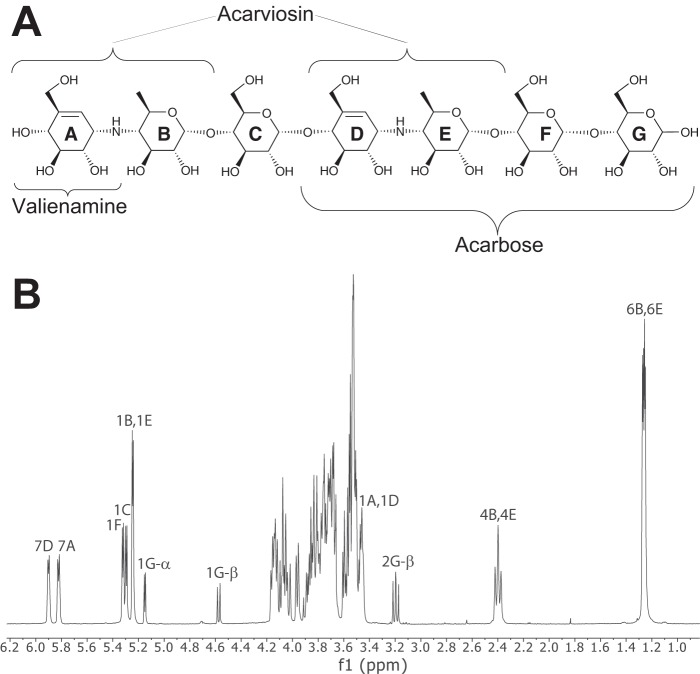
**Characterization of acarviostatin II01 by ^1^H NMR.** *A*, structure of acarviostatin II01; *B*, its one-dimensional ^1^H NMR spectrum (400 MHz, D_2_O, 25 °C). Partial signal assignment was made using two-dimensional COSY, HSQC, and ROESY NMR experiments and based on a previous detailed assignment of acarviosin-containing oligosaccharides (59).

AtDPE1 shares 75% amino acid sequence identity with the equivalent enzyme from potato, for which there is a crystal structure available (PDB code 1X1N).^5^ This was used as a monomer template to solve the ligand-free (LF) AtDPE1 structure at 2.13 Å resolution by molecular replacement in PHASER (35). Two copies of the template were placed in the asymmetric unit, giving a homodimer equivalent to that deduced as the biological unit for the template structure. This was consistent with previous gel filtration results, which showed that AtDPE1 was dimeric in solution (11). The model of the LF structure was completed through several iterations of refinement with REFMAC5 (36) and model building with COOT (37). The final LF model was used as a starting point for the modeling and refinement of the structures of the ligand-bound complexes. The quality of the structures was evaluated using MolProbity (38), and the statistics of the final models are summarized in Table 2. All structural figures were prepared using CCP4mg (39). For convenience, the AtDPE1 complex structures are referred to by three-letter abbreviations of the compound used for the soaking, and when discriminating between monomers in the same structure, an A or B suffix is used (*e.g.* ACR-A and ACR-B refer to the A and B subunits, respectively, in the structure determined from the acarbose-soaked crystal). Where ligand binding was comparable between the two chains for a given structure, only the A chain was described (*e.g.* ACV-A and BCD-A). Although it was not always possible to precisely define the stereo- and regiochemistry of the sugars, in general, the electron density was consistent with sugars in ^4^C_1_ chair conformations, with the exception of the valienamine residues (defined in Fig. 1*A*) derived from acarbose, which were built in ^2^H_3_ half-chair conformation. Also, all of the glycosidic linkages were modeled as α-1,4, apart from the covalent bonds to the active site nucleophile, which were modeled as β linkages.

**TABLE 2:** **Refinement of x-ray structures**

Data set	LF	MA3	ACR	ACV	BCD	CAM
Resolution range (Å)*^a^*	57.78–2.13 (2.19–2.13)	63.74–1.80 (1.85–1.80)	63.67–2.05 (2.10–2.05)	40.95–2.53 (2.60–2.53)	63.63–2.30 (2.36–2.30)	32.03–2.30 (2.36–2.30)
Reflections: working/free*^b^*	64,404/3459	100,068/5261	68,519/3689	39,812/2181	49,836/2692	50,050/2688
Final *R*_work_*^a,c^*	0.187 (0.309)	0.153 (0.223)	0.187 (0.299)	0.207 (0.276)	0.163 (0.244)	0.176 (0.250)
Final *R*_free_*^a,c^*	0.218 (0.324)	0.179 (0.270)	0.210 (0.313)	0.249 (0.376)	0.196 (0.272)	0.205 (0.301)
Cruickshank DPI (Å)	0.177	0.102	0.161	0.298	0.193	0.202
r.m.s.*^d^* bond deviations (Å)	0.012	0.012	0.012	0.010	0.010	0.011
r.m.s. angle deviations (degrees)	1.40	1.43	1.49	1.44	1.31	1.39
No. of protein residues in chain A, chain B (ranges)	512 (59–328, 335–576), 514(59–329, 334–576)	517 (60–576), 528(60–328, 334–576)	506 (60–384, 396–576), 517(60–576)	518 (59–576), 518(59–576)	514 (60–328, 332–576), 511(60–328, 335–576)	514 (60–328, 332–576), 511(60–328, 335–576)
No. of heterogen residues: sugar/water/ethylene glycol	0/535/14	17/687/16	14/403/11	14/200/3	4/333/16	5/309/16
Mean *B*-factors: protein/sugar/water/ethylene glycol/overall (Å^2^)	43/-/42/50/43	32/44/40/37/33	45/56/43/55/45	43/49/40/62/44	41/52/41/51/41	42/70/39/51/42
Ramachandran plot: favored/allowed/disallowed (%)*^e^*	97.7/2.3/0.0	98.8/1.2/0.0	98.1/1.7/0.2	97.4/2.6/0.0	98.6/1.2/0.2	98.5/1.5/0.0
PDB accession code	5CPQ	5CPS	5CSY	5CSU	5CPT	5CQ1

*^a^* Values for the outer resolution shell are given in parentheses.

*^b^* The data set was split into “working” and “free” sets consisting of 95 and 5% of the data, respectively. The free set was not used for refinement.

*^c^* The *R*-factors *R*_work_ and *R*_free_ are calculated as follows: *R* = Σ(|*F*_obs_ − *F*_calc_|)/Σ|*F*_obs_| × 100, where *F*_obs_ and *F*_calc_ are the observed and calculated structure factor amplitudes, respectively.

*^d^* r.m.s., root mean square.

*^e^* As calculated using MOLPROBITY (38).

##### Amino Acid Sequence Numbering

The wild-type AtDPE1 sequence bears an N-terminal extension relative to the thermophilic bacterial amylomaltases (26), requiring one to add roughly 80 to the numbering scheme used for the latter enzymes in order to identify corresponding residues in AtDPE1. The first 45 residues of the AtDPE1 sequence constitute the transit peptide responsible for organellar targeting, and this was not present in the construct used for crystallization.

##### Production of Acarviostatin II01

**FIGURE 2.**
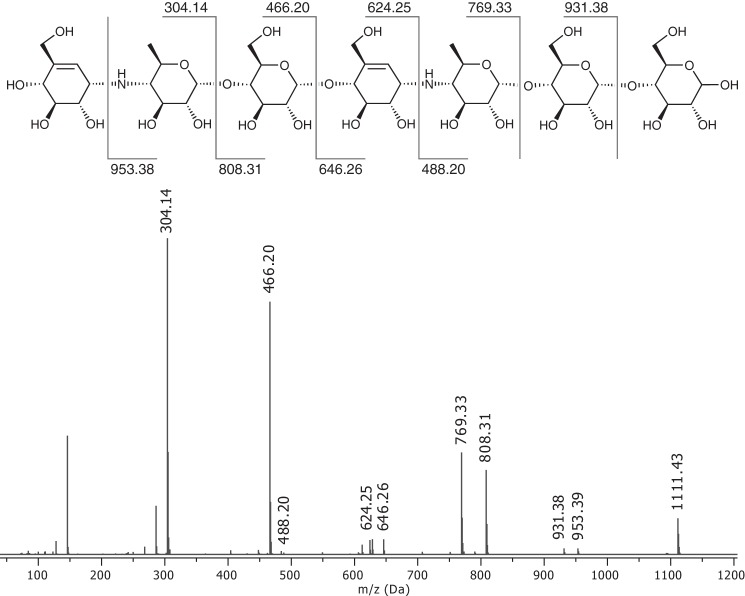
**Positive ion mode ESI-MS/MS fragmentation of acarviostatin II01 [M + H]^+^ at *m*/*z* 1111.43.**

Acarbose (100 mg) was dissolved in 5 ml of aqueous triethylammonium acetate (50 mm), and 10 μl of AtDPE1 (20 mg/ml) was added to the solution. The reaction was incubated at room temperature for 2 weeks with additional aliquots of the enzyme (10 μl each) added every 4 days. The enzyme was removed by spin filtration (10,000 molecular weight cut-off; Millipore), and acarviostatin II01 (40) was purified by HPLC on a Luna NH_2_ column (5-μm NH_2_, 100 Å, 250 × 10 mm; Phenomenex Inc.) using a Dionex HPLC Ultimate 3000, coupled with a Dionex charged aerosol detector and a Foxy Jr. fraction collector (Teledyne Isco Inc.). The mobile phase was a gradient of acetonitrile, from 90 to 55% over 35 min, followed by H_2_O for 10 min and 90% acetonitrile for 10 min, at a flow rate of 5 ml/min. The fractions collected were analyzed by thin layer chromatography to identify those containing acarviostatin II01 (41). TLC Silica Gel 60 F_256_ (Merck) was used as the stationary phase, and CH_3_CN/H_2_O/NH_4_OH at a ratio of 6:3:1 was used as the mobile phase. The compounds were visualized by dipping the plate in 0.5% (w/v) orcinol in 20% HCl (v/v in ethanol) followed by heating. Fractions containing acarviostatin II01 (Fig. 1*A*) were pooled and freeze-dried to afford a white solid (2.3 mg, 1.34%). *R_f_* = 0.11 (silica gel, CH_3_CN/H_2_O/NH_4_OH 6:3:1). ^1^H NMR (400 MHz, D_2_O): δ_H_ 5.90 (1 H, d, *J* 4.0 Hz, H7-D), 5.82 (1 H, d, *J* 4.8 Hz, H7-A), 5.32 (1 H, d, *J* 3.9 Hz, H1-F), 5.29 (1 H, d, *J* 3.9 Hz, H1-C), 5.24 (2 H, m, H1-B, H1-E), 5.15 (1 H, d, *J* 3.7 Hz, H1-Gα), 4.57 (1 H, d, *J* 8.0 Hz, H1-Gβ), 4.18–3.44 (m), 3.19 (1 H, dd, *J* 9.5 Hz, *J* 8.0 Hz, H2-Gβ), 2.40 (2 H, m, H-4B, H-4E), 1.26 (6 H, dd, *J* 6.3 Hz, *J* 2.9 Hz, H-6B, H-6E) (Fig. 1*B*). ESI-MS/MS(+) at *m/*z 1111.43 ([M + H]^+^); 304.14, 466.20, 488.20, 624.25, 646.26, 769.33, 808.31, 931.38, 953.39 (Fig. 2). HR-ESI-MS(+): *m/z* Calcd [C_44_H_74_N_2_O_30_]H^+^: 1111.4399. Found: 1111.4406.

## Results

### 

#### 

##### Structural Overview of AtDPE1

**FIGURE 3.**
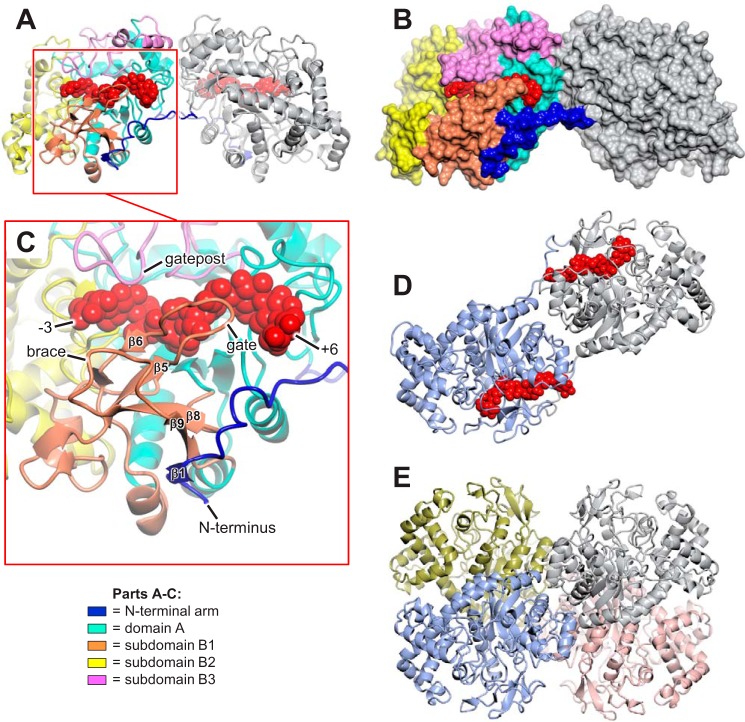
**Structural summary of AtDPE1.** *A*, the elongated AtDPE1 homodimer is shown in a schematic representation. The N-terminal arms of both subunits are *colored* in *dark blue*; otherwise, the *left-hand* subunit is *colored* by domain as indicated (see also Fig. 4), and the *right-hand* subunit is in *gray*. The structure shown is that derived from a crystal soaked in maltotriose (MA3) with maltononaose bound to the *left-hand* subunit and maltooctaose bound to the *right-hand* subunit, depicted as *red van der Waals spheres. B*, same view as in *A* but with the protein represented as a molecular surface. This clearly shows how the N-terminal arm of the *right-hand* subunit embraces its partner and contributes significantly to the dimer interface. *C*, *close-up* taken from *A* focusing on the region containing the active site canyon, which is defined by loops from the B1, B2, and B3 subdomains. The elements of the antiparallel β-sheet made up of four strands from subdomain B1 and one from the N-terminal arm of the opposing subunit are *labeled*; β5 and β6 delineate the gate motif, whereas β8 and β9 delineate the brace motif. *N terminus*, the first crystallographically resolved residue (Ser-60). *D*, AtDPE1 MA3 structure viewed from above relative to the perspective shown in *A* but with the *left-hand* subunit in *slate blue. E*, the homotetrameric amylomaltase from *A. aeolicus* (PDB code 1TZ7) *arranged* and *colored* to highlight the observation that it resembles a pair of intertwined AtDPE1 dimers (note the similarity with the dimer in *D*); such an assembly is not possible in AtDPE1 due to steric clashes with the N-terminal arms.

**FIGURE 4.**
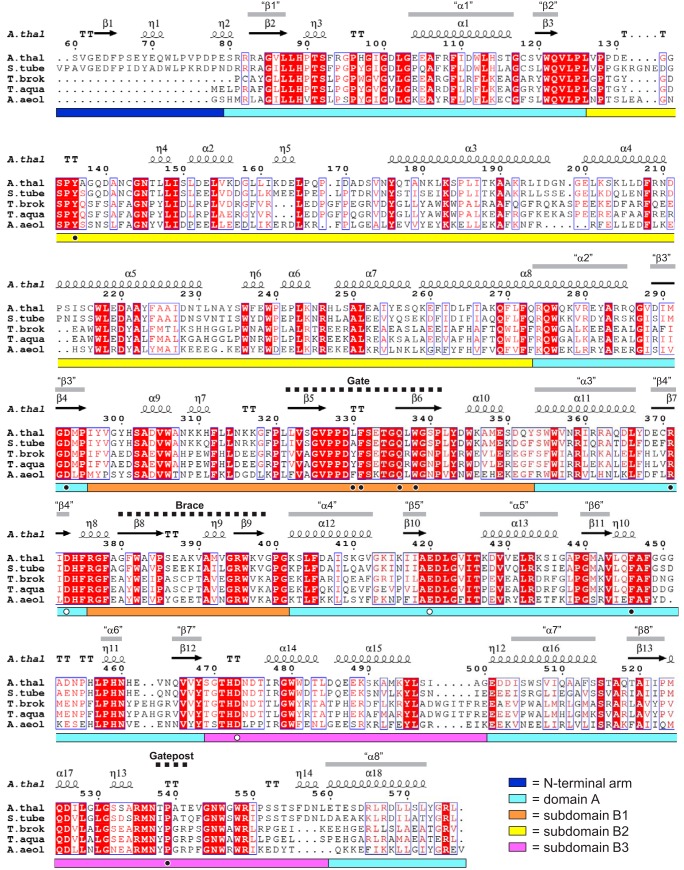
**Structure-based multiple-sequence alignment of AtDPE1 with its closest structural homologues.** Shown are the sequences of DPE1 enzymes from *A. thaliana* (*A.thal*; MA3-A structure) and *S. tuberosum* (*S.tube*; PDB code 1X1N) and amylomaltases from *T. brokianus* (*T.brok*; PDB code 2X1I), *T. aquaticus* (*T.aqua*; PDB code 1ESW), and *A. aeolicus* (*A.aeol*; PDB code 1TZ7). The sequence for the *T. thermophilus* enzyme was not included because this differs from that of *T. aquaticus* by only a single amino acid (at position 104 in AtDPE1 numbering). The initial alignment was generated using the PDBeFold server (60). This was subsequently adjusted manually with reference to the superposed structures and then displayed using ESPript3.0 (61). Strictly conserved residues are highlighted with *red shaded boxes*, and semiconserved residues are *colored red*. Secondary structure elements for AtDPE1 are shown *above* the alignment, where α = α helix, β = β strand, η = 3_10_ helix, TT = β turn. Also labeled are the secondary structural elements of the central (βα)_8_ barrel (*i.e.* domain A) as “β*1*,” “α*1*,” etc., and the positions of the gate, brace, and gatepost motifs. The *colored bars below* the alignment denote the structural domains as indicated in the *key* and displayed in Fig. 3. The *white dots* show the positions of the three catalytic residues, and the *black dots* indicate other important residues that are referred to throughout.

The AtDPE1 crystal structure reveals an elongated homodimer some 120 Å in length (Fig. 3). Each protomer contains the canonical (βα)_8_ barrel fold (42) expected at the core of a GH77 family enzyme (23). This is referred to as domain A (Figs. 3 and 4), following the nomenclature used by Przylas *et al.* (26). The latter is decorated by several extended loops that arise from the C-terminal ends of five of the eight parallel β-strands of the barrel. These form three subdomains, B1–B3 (26), that collectively define the walls of a deep canyon, running perpendicular to the barrel axis and open at both ends. Through comparisons with related enzymes (described below; Table 3), the catalytic residues are predicted to be Asp-373 (the nucleophile) and Glu-420 (the acid/base catalyst), lying at the ends of the 4th and 5th β-strands (β4 and β5 in Fig. 4), respectively, of domain A and positioned at the base of the canyon, where they mark the boundary between the donor and acceptor substrate binding sites.

**TABLE 3:** **Selected structural homologues of AtDPE1**

Protein	Source	Biological unit	Ligand bound	PDB code*^a^*	Resolution	DALI output			r.m.s.*^c^* deviation (Å)/aligned residues	Interface area*^d^*	Source/Reference
						Rank*^b^*	*Z*-Score	Identity			
					Å			%		Å^2^	
DPE1	*A. thaliana*	Dimer	Malto-nonaose	5CPS	1.80			100	0.00/517	2006	This work
DPE1	*S. tuberosum*	Dimer	No	1X1N	1.80	1	55.3	75	1.03/511	2380	Imamura *et al.**^e^*
Amylomaltase	*T. brockianus*	Monomer	No	2X1I	2.36	2	49.8	42	1.42/466		Ref. 28
Amylomaltase	*T. aquaticus*	Monomer	ACR	1ESW	1.90	3	49.6	41	1.41/475		Ref. 25
Amylomaltase	*T. thermophilus*	Monomer	ACR and 4-deoxyglucose	2OWW	2.20	3	49.2	42	1.56/476		Ref. 27
Amylomaltase	*A. aeolicus*	Tetramer	No	1TZ7	2.15	4	49.2	43	1.44/460	1147/1416*^f^*	Barends *et al.**^g^*
Amylomaltase	*E. coli*	Monomer	ACR	4S3R	2.10	5	33.2	21	2.17/447		Ref. 29
GlgE	*S. coelicolor*	Dimer	Maltose	3ZT5	2.10	6	24.4	13	2.49/262	2168*^h^*	Ref. 44
Cyclodextrin glycosyl-transferase	*P. macerans*	Monomer	No	4JCL	1.70	10	22.0	12	2.60/250		Wu *et al.**^i^*

*^a^* Results of a DALI search (62) using MA3-A structure. The hits were filtered for redundancy; where a relevant ligand bound structure exists for a particular enzyme, this is the one that is shown.

*^b^* Ranking of the DALI hit (after redundancy filtering at 90% sequence identity). The sequences of the *T. aquaticus* and *T. thermophilus* enzymes differ by only a single amino acid residue and thus have been given equal ranking. Hits 7–9 are bacterial α-amylases, and hit 10 is the closest cyclodextrin glycosyltransferase.

*^c^* r.m.s., root mean square deviation.

*^d^* Calculated using PDBePISA (63).

*^e^* K. Imamura, T. Matsuura, T. Takaha, K. Fujii, A. Nakagawa, M. Kusunoki, and Y. Nitta, unpublished results.

*^f^* There are two non-equivalent types of intersubunit interfaces within the tetramer of *A. aeolicus* amylomaltase. The first value given corresponds to the dimer interface seen in the DPE1 structures; this is significantly lower because this enzyme lacks the N-terminal arm characteristic of the plant enzymes.

*^g^* T.R.M. Barends, H. Korf, T. Kaper, M.J.E.C. van der Maarel, L. Dijkhuizen, and B.W. Dijkstra, unpublished results.

*^h^* Does not correspond to the DPE1 dimer interface.

*^i^* L. Wu, J. Zhou, J. Wu, J. Li, and J. Chen, unpublished results.

**FIGURE 5.**
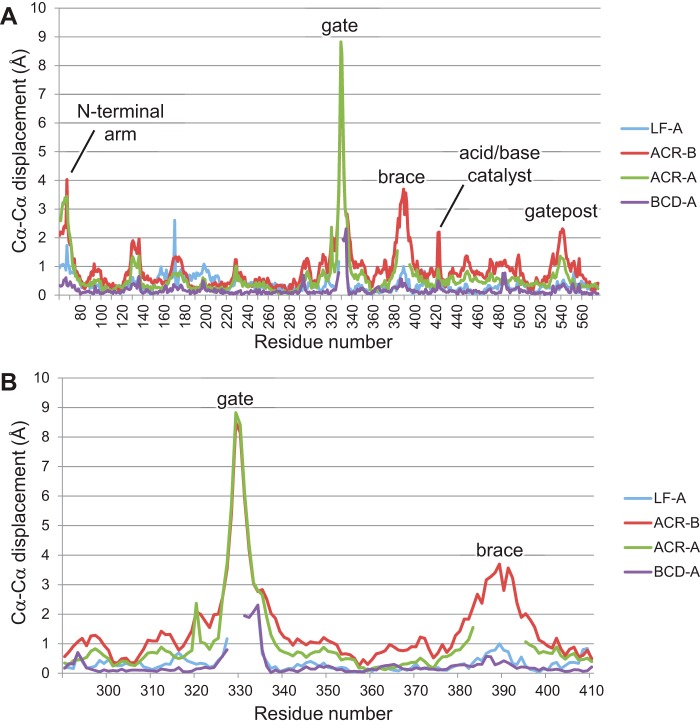
**Plots highlighting conformational changes between selected AtDPE1 structures.** *A*, Cα-Cα displacements relative to the MA3-A structure are plotted for the whole sequence; *B*, the plot is expanded to focus on just the region encompassing the whole of the B1 subdomain. Gaps indicate disordered regions.

In the various AtDPE1 structures presented herein, the most significant conformational changes are restricted to the N terminus and the B1 and B3 subdomains (Figs. 3 and 5). The B1 subdomain provides one wall of the substrate-binding canyon and is composed of two insertions in the (βα)_8_ barrel, both of which contain a β-hairpin; these abut one another to form an anti-parallel four-stranded β-sheet. The loops defined by the β-hairpins display the most conformational flexibility. We shall refer to these as the “gate” (equivalent of the 250s loop in amylomaltases (26)), which directly flanks the canyon, and the “brace,” which packs against the base of the gate, respectively (Fig. 3*C*). The opposing canyon wall is largely made up of subdomains B2 and B3. In the latter, an extended loop (equivalent of the 460s loop in amylomaltases) partially projects over the donor binding site and is also mobile but to a lesser extent; we shall refer to this as the “gatepost.”

Ser-60 is the first residue that can be clearly resolved in the electron density, and the ensuing segment up to and including Glu-79 forms an extended arm that embraces the opposing subunit in the homodimer (Fig. 3) and makes a significant contribution to the dimer interface; the buried surface area totals some 2,000 Å^2^, as calculated by PISA (available on the PDBePISA Web site) (43). Moreover, residues 63–65 form a short β-strand that extends the β-sheet of the B1 subdomain in the opposing subunit by packing against the aforementioned brace (Fig. 3*C*). By contrast, the C-terminal residue, Leu-576, is fully resolved in all of the AtDPE1 structures.

Further discussion of the subunit topology is not warranted here because this has been adequately described elsewhere for the thermophilic bacterial amylomaltases (26–28), with which AtDPE1 is closely superposable (see below and Table 3).

##### Structural Homologs of AtDPE1

Interrogation of the Protein Data Bank using the DALI server retrieves 117 non-redundant entries with significant structural similarity to AtDPE1 (*Z* scores >10.0). Of these, five proteins stood out as being extremely similar to AtDPE1 (*Z* scores ∼50); these were the potato DPE1 (*Solanum tuberosum* DPE1; PDB code 1X1N),^5^ and four amylomaltases from thermophilic bacteria, namely *Thermus brokianus* (PDB code 2X1I) (28), *Thermus aquaticus* (PDB code 1ESW) (25), *Thermus thermophilus* (PDB code 2OWW) (27), and *Aquifex aeolicus* (PDB code 1TZ7),^6^ respectively (Table 3). The next hit was MalQ, the mesophilic amylomaltase from *E. coli* (*Z* score 33.2; PDB code 4S3R) (29). Hit number 6 was the maltosyltransferase GlgE (Z score 24.4; PDB code 3ZT5) (44), which has a gate structurally equivalent to that of AtDPE1, but the corresponding loop is much shorter in GlgE, such that it only partially covers the donor site (not shown). Moreover, the donor site is a dead-end in GlgE, where it is open-ended in AtDPE1. The first DALI hit annotated as a cyclodextrin glycosyltransferase was number 10 (*Z* score 22.0; PDB code 4JCL).^7^ Similar to the amylomaltases and DPE1, cyclodextrin glycosyltransferases also produce cyclic products, but these are the smaller cyclodextrins (DP 6–8). Notably, cyclodextrin glycosyltransferases do not have the gate, and it has been proposed previously that the latter might prevent the formation of small cyclic products in amylomaltases through steric hindrance (26). Whereas AtDPE1 and *S. tuberosum* DPE1 form comparable homodimers, four of the amylomaltases are monomeric, and the fifth, *A. aeolicus* AMY, is homotetrameric (Table 3). One of the interfaces in this latter structure corresponds to that seen in the plant dimers, such that it resembles a pair of intertwined plant enzyme dimers (Fig. 3, *D* and *E*). Assembly of such a tetramer is not possible for the plant enzymes because the formation of the additional interfaces would be precluded by steric clashes with the N-terminal arms. Finally, from these comparisons, it is clear that the gate motif is a defining feature of the DPE1 enzymes and the amylomaltases. Its importance is reflected by a high degree of sequence conservation within the enzyme family; across AtDPE1 and its closest five structural homologs, the 20-residue β-hairpin that forms the gate (residues 322–341 inclusive in AtDPE1) contains 14 strictly conserved residues (Fig. 4). Nine of these are also conserved in *E. coli* AMY (not shown).

##### Comparison of AtDPE1 Structures

**FIGURE 6.**
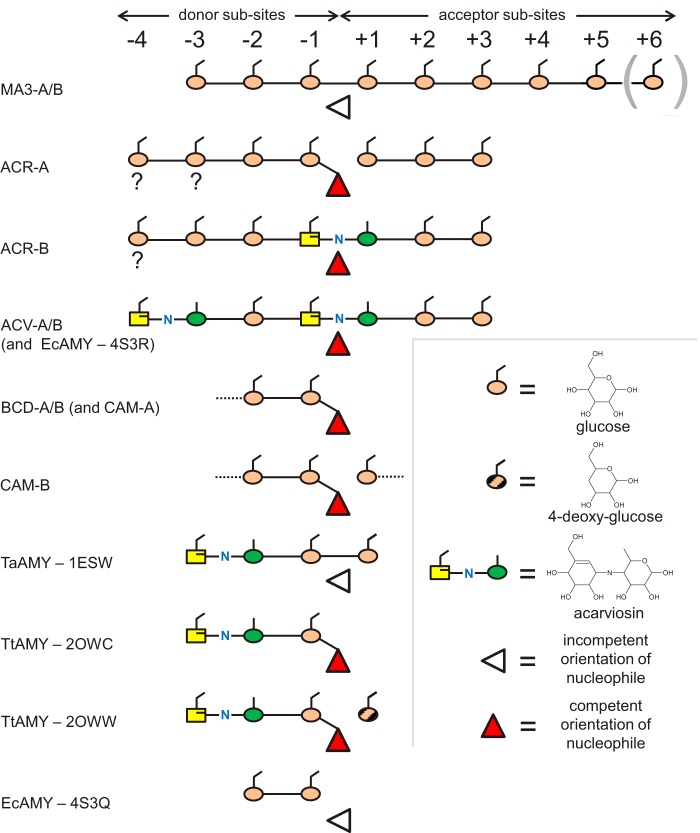
**Schematic representations of the ligand-bound complexes of AtDPE1.** For each different binding mode, the occupancy of the various subsites is indicated. The *triangles* indicate the status of the active site nucleophiles. For MA3-A, density was present for a residue in the +6 site, but not for MA3-B; otherwise, the bound ligands were very similar. For ACR-A and ACR-B, the identities of several of the sugar residues were uncertain (indicated by the *question marks below* these residues), and a non-glucose sugar was only inserted when there was compelling evidence for it. Also shown for comparison are the relevant ligand-bound complexes of the amylomaltases.

In this study, in addition to the structure of LF AtDPE1, we determined the structures of five ligand-bound complexes of the enzyme. Moreover, for some of these, different binding modes were observed between the two active sites within the crystallographic asymmetric unit, giving rise to a total of six discrete ligand-bound states (Fig. 6).

##### AtDPE1 Maltotriose Soak

**FIGURE 7.**
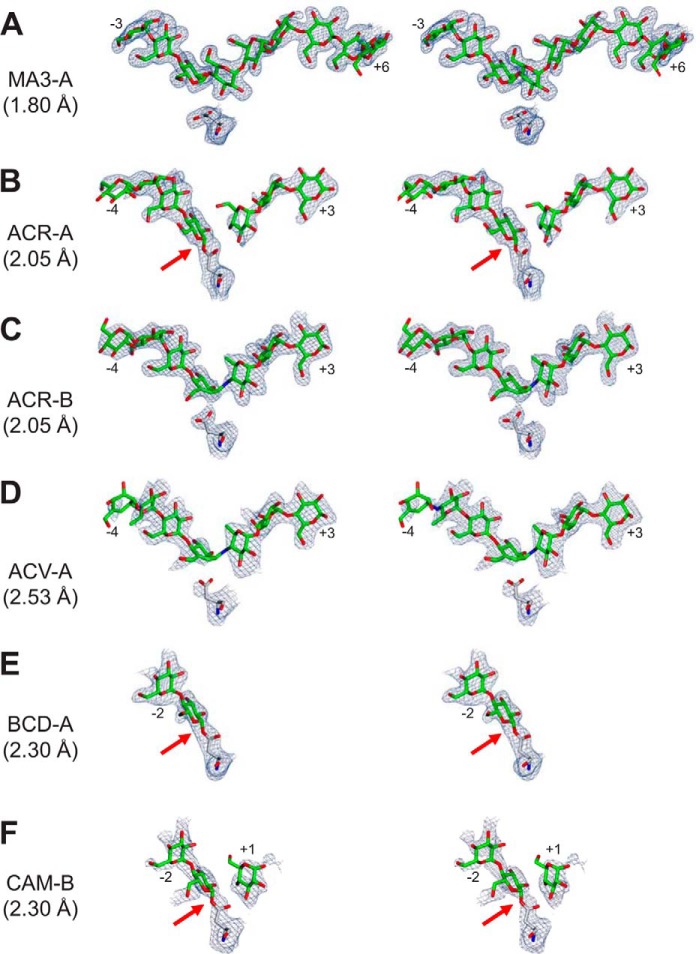
**Stereoviews showing omit electron density maps for ligands bound to AtDPE1.** Omit electron density maps were calculated after removal of the bound ligands and the catalytic nucleophile (Asp-373) and re-refining. These are displayed contoured at ∼3σ superimposed on the refined coordinates of the atoms that were omitted. The resolutions of each of these are indicated. Where the ligand occupying the donor site is covalently linked to the nucleophile, this has been highlighted with a *red arrow*.

**FIGURE 8.**
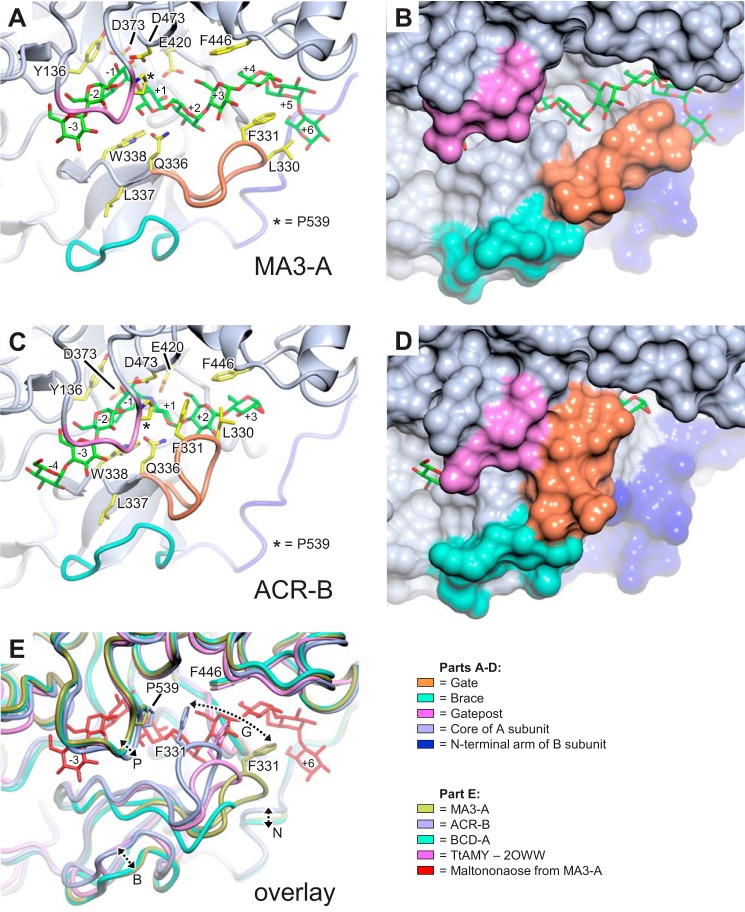
**Conformational changes coupled to active site occupancy in AtDPE1.** Overview of conformation changes between MA3-A (*A* and *B*) and ACR-B (*C* and *D*) structures, where the protein backbone is either shown in a schematic representation (*A* and *C*) or displayed as a molecular surface (*B* and *D*); selected key residues and the bound ligands are shown in *stick representations. E*, overlay of MA3-A, ACR-B, and BCD-A AtDPE1 structures with *T. thermophilus* AMY (PDB code 2OWW) to illustrate conformational differences between the gate (*G*), brace (*B*), gatepost (*P*), and N-terminal arm (*N*), which are highlighted by the *double-headed dashed arrows*. In ACR-A and ACV-A/B, these motifs display conformations similar to that of ACR-B (not shown), although the brace was not fully ordered in ACR-A (see Fig. 5). Note that the gate in BCD-A displays the most open conformation but is disordered at the tip, such that Phe-331 is not resolved in the electron density.

**FIGURE 9.**
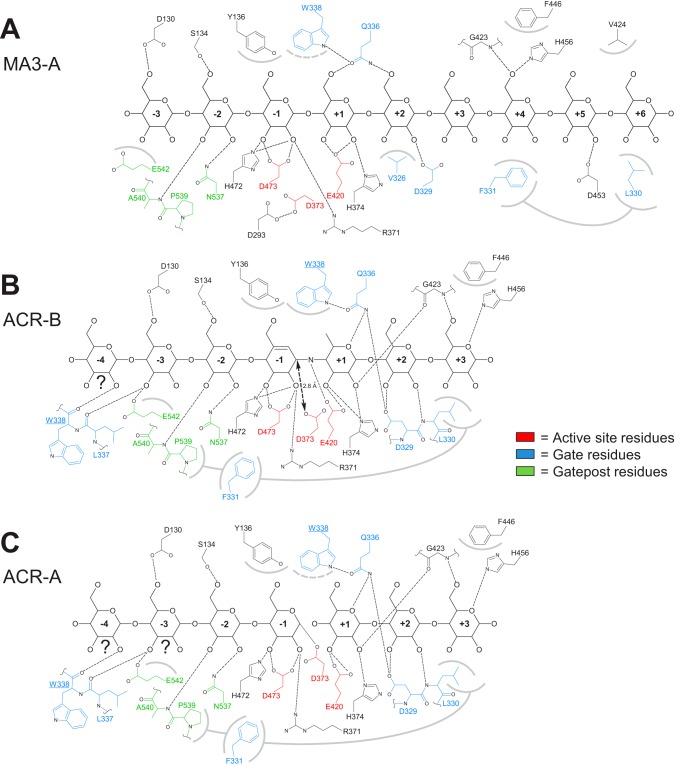
**Schematic figures detailing protein-ligand interactions for selected AtDPE1 complexes.** Shown are protein-ligand interactions for MA3-A (*A*), ACR-B (*B*), and ACR-A (*C*), where *dashed lines* indicate hydrogen bonds and *gray arcs* indicate van der Waals interactions with the adjacent sugar(s). The *connecting gray lines* indicate interactions between protein side chains. For simplicity, hydrogens have been omitted as well as any water-mediated interactions. In *B* and *C*, Trp-338 makes both hydrogen bonding and hydrophobic interactions with sugars that are widely spaced in this two-dimensional representation, and for this reason, the residue appears twice. For ACR-A and ACR-B, the identities of several of the sugar residues were uncertain (indicated by the *question marks below* these residues).

**FIGURE 10.**
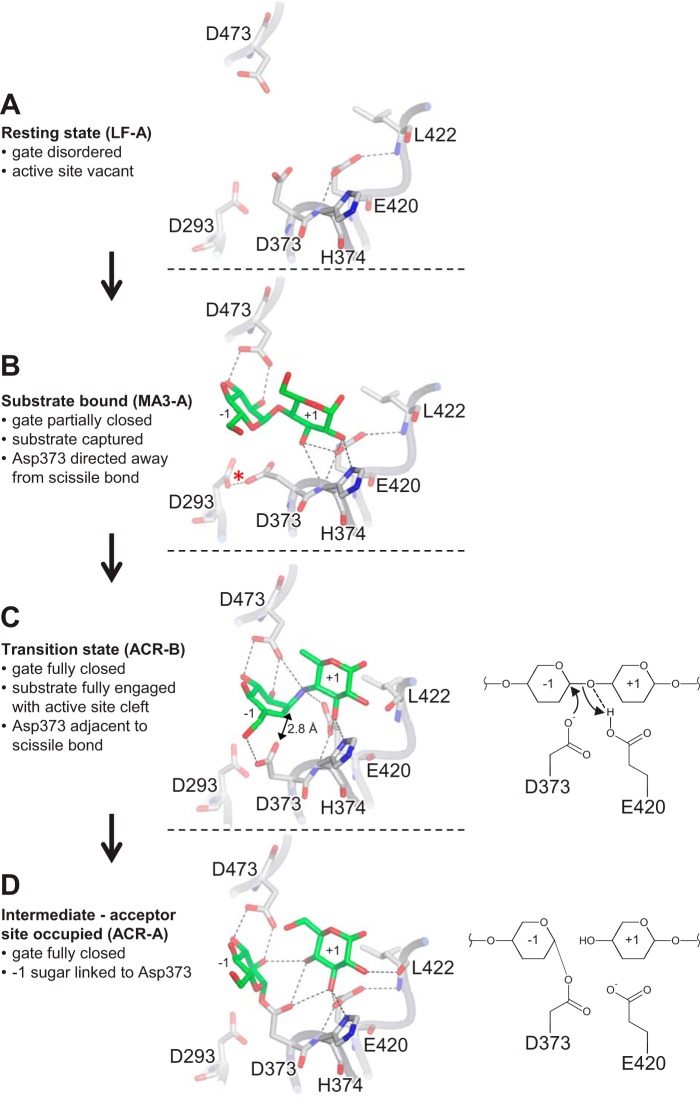
**Detail of important interactions at the active site of AtDPE1 relevant to catalysis.** Selected key amino acids are shown in *stick representation* at the junction of donor and acceptor binding sites. Hydrogen bonds are indicated by *dashed lines*, with the putative low barrier hydrogen bond (referred to under “Discussion”) marked by a *red asterisk* in *B*. For each part, the coordinates are taken from the structures given in *brackets*, and for the ligand-bound structures only the −1 and +1 sugars are shown for simplicity. For *C* and *D*, a schematic representation is also shown to the *right* to illustrate the progression from the transition state to the intermediate (for clarity, the sugars are shown as simple six-membered rings). The incompetent conformation of the nucleophile (Asp-373) in *B* may be more representative of a post-catalytic product-bound state (where a further catalytic event would be counterproductive) and may be dictated by the occupancy of the acceptor site (note that subsites +2 to +6 are also occupied in MA3-A but are not shown here). However, in a precatalytic substrate-bound state with maltotriose spanning subsites −2 to +1, where catalysis is desirable, there would be no necessity for Asp-373 to adopt the incompetent conformation. In the absence of such a structure, this remains just speculation.

The MA3 structure of AtDPE1 was determined to 1.8 Å resolution and revealed clear density for a malto-oligosaccharide in each active site, indicative of transglycosylation reactions occurring during crystal soaking (Fig. 7). In subunit A (MA3-A), nine glucose residues could be resolved in the density, spanning some 33 Å and occupying both the donor and acceptor sites, specifically subsites −4 to +6 (Figs. 3, 6, and 8*A*), whereas eight were resolved for the B chain (MA3-B). The glucan chains adopt a helical structure consistent with that observed for α-1,4-linked glucans in solution (45), although it is overtwisted at the junction between donor and acceptor sites. The bound oligosaccharides make extensive interactions with the protein (Fig. 9*A*), especially so around the expected site of cleavage, where the residues involved are conserved in the close structural homologues (Fig. 4). The sugar in the −1 donor subsite, in particular, has a bidentate hydrogen bonding interaction through O2 and O3 with Asp-473, a configuration that is seen throughout the α-amylase family. Indeed, this acid is often referred to as the “transition state stabilizer” for reasons that will be discussed below. Additionally, the −1 sugar stacks against Tyr-136, previously described as a “platform residue” (28), but has a much looser association with the side chain of Trp-338 that approaches from the opposite face. A further important interaction is made between the sugar in the +1 acceptor site and Gln-336; it is notable that Gln-336 and Trp-338 both lie at the base of the gate. In the LF structure, the central portion of the gate is disordered. By contrast, in MA3-A the gate is fully ordered and adopts a partially closed conformation. This allows the clamping of the +3 and +4 sugars between the hydrophobic “jaws” of Phe-331, lying at the tip of the gate, and Phe-446 from the opposite side of the substrate binding cleft, which sits at the end of the sixth β-strand (β6) of domain A (Figs. 4, 8 (*A* and *B*), and 9*A*). Consistent with the mobility of the gate, the electron density is weaker in this region, and the refined temperature factors are elevated relative to the core of the structure. Indeed, in MA3-B, the density is weaker still, such that the end of the gate cannot be confidently modeled. Despite the strict conservation of Phe-331 and Phe-446 in the closest structural homologues, this interaction has not been observed before due to the lack of a suitable ligand-bound structure with the +3 and +4 sites occupied; it has, however, been predicted (28). The conformation of the brace is essentially unchanged between the LF and MA3 structures (Fig. 5). Although this complex seems poised for catalysis, the ligand is not fully engaged with the active site (compared with complexes described below), and one key residue, Asp-373, the presumed catalytic nucleophile, is directed away from the scissile bond due to a ∼115° rotation around the Cα–Cβ bond relative to its conformation in the LF structure (Fig. 10, *A* and *B*). The potential significance of this catalytically “incompetent” configuration will be discussed further below. In many respects, this structure resembles the non-covalent complexes of *T. aquaticus* AMY with acarbose (PDB code 1ESW) (25) and *E. coli* AMY and maltose (PDB code 4S3Q) (29), respectively, where the ligands are also not fully engaged, and the catalytic nucleophiles adopt the same incompetent configuration (Fig. 6).

##### AtDPE1 Acarbose Soak

When a crystal was soaked in the pseudotetrasaccharide inhibitor acarbose, the active sites of the resultant 2.05 Å resolution structure (ACR) revealed two distinctly different binding modes, both of which required modification of the ligand (Fig. 7, *B* and *C*); this has previously been seen crystallographically for other enzymes of the GH-H clan (46–48), including *E. coli* AMY (29), where the observed ligands can be explained by transglycosylation events that link two or more acarbose molecules with the concomitant loss of glucose from the reducing end of the donor molecule. In ACR-B, the density indicated the presence of a heptasaccharide spanning subsites −4 to +3. The density for the −1 sugar was indicative of a ^2^H_3_ half-chair conformation, and for the +1 sugar, there was no evidence for a hydroxyl at C6. Therefore, this was consistent with an acarbose molecule occupying subsites −1 to +3, placing the non-cleavable bond at the junction of the donor and acceptor sites (Fig. 6). From the density in subsites −4 to −2, it was not possible to confidently assign anything other than glucose units in ^4^C_1_ chair conformation. Thus, maltotriose was inserted here attached to the non-reducing end of the acarbose via an α-1,4-linkage (Fig. 6). Although it is difficult to rationalize the creation of such a ligand through transglycosylation alone, there are structural precedents for similar products forming upon the incubation of acarbose with α-amylases (49–51) as well as enzymatic pathways postulated for their production (51, 52). Compared with the nonasaccharide in MA3-A, the heptasaccharide in ACR-B is more deeply buried in the active site cleft, so that the positions of the subsites are slightly different. Nevertheless, many of the interactions with the protein are conserved (Fig. 9, *A* and *B*), mainly due to a concomitant repositioning of side chains, including all of the aforementioned interactions with the −1 sugar, although here the side chain of Trp-338 is now closely associated with this sugar, such that the latter is more firmly clamped between this residue and Tyr-136 (Fig. 8*C*). In this complex, the distorted conformation of the valienamine moiety at subsite −1 (Fig. 10*C*) is thought to mimic the oxocarbenium-like transition state expected for enzymes of the α-amylase family (53). Indeed, the bidentate interaction with Asp-473 is expected to play a dual role in lowering the energy of the transition state by stabilizing this half-chair conformation and by neutralizing the positive charge of the oxocarbenium ion. A further, crucial, difference with the MA3-A structure is that the side chain of Asp-373 is appropriately oriented for catalysis with its carboxylate (O^δ1^) just 2.8 Å from the C1 atom of the −1 sugar (Figs. 9*B* and 10*C*). At the same time, more subtle changes to the conformation of Glu-420 enable its carboxylate group (O^ϵ2^) to hydrogen-bond to the nitrogen atom of the acarviosin moiety (defined in Fig. 1*A*). Finally, in ACR-B, the gate swings over the active site canyon to adopt a new, fully closed conformation, whereby Phe-331 now stacks against Pro-539 in the gatepost (Figs. 8 (*C* and *D*) and 9*B*), the latter having moved relative to its position in MA3-A (Figs. 8 (*A* and *B*) and 9*A*). The side chain of Phe-331 moves by over 11 Å (relative to its position in MA3-A) and, in so doing, shields the active site from bulk solvent. A more subtle, but nevertheless significant, movement is seen in the brace, which moves in conjunction with the gate (Fig. 8*E*). As a consequence of the gate movement, the sugars in the acceptor site are no longer clamped between the Phe-331/Phe-446 aromatic pair. Nevertheless, Phe-446 still serves as a platform for the substrate but is now stacking mainly with the +3 sugar (Figs. 8*C* and 9*B*).

By contrast, in ACR-A, although the gate is also fully closed, and there is also density for sugars occupying all of subsites −4 to +3, the density is broken between the −1 and +1 sugars (Fig. 7*B*). Instead, there is continuous density between the reducing end of the −1 sugar and Asp-373, indicating that this represents an intermediate state with a covalently bound substrate in the donor site linked to the O^δ2^ atom of Asp-373 via a β-glycosidic bond (Figs. 6, 9*C*, and 10*D*). Given the lack of clear evidence for non-glucose residues in any of the subsites, for simplicity, all of the sugars were modeled as α-1,4-linked glucose units in ^4^C_1_ chair conformation (Fig. 6). With the exception of the −2 and −1 sugars, the remaining sugars superposed well between ACR-A and ACR-B. Also, in ACR-A, the electron density for the brace was indicative of more than one conformation and showed some correspondence both to the position seen with a partially closed gate (as in MA3-A) and to the position seen with a fully closed gate (as in ACR-B), but neither conformation (nor a mixture of the two) could be satisfactorily modeled and refined. Therefore, this region (residues 385–395 inclusive) was omitted from the final coordinates. Closer inspection of the density for the gate in ACR-A (which was weaker than that for ACR-B) revealed some evidence for a partially closed conformation, but this could not be modeled with confidence. The final refined positions of the gate in ACR-A and ACR-B were essentially identical, both being fully closed (Fig. 5). In comparisons with homologous structures, ACR-A most closely resembles the covalent complexes of acarbose with *T. thermophilus* amylomaltase AMY (PDB codes 2OWC and 2OWW) (27).

##### AtDPE1 Acarviostatin II01 Soak

A crystal soaked in the heptasaccharide mimic acarviostatin II01 diffracted to 2.53 Å resolution, and the resultant structure (ACV) revealed essentially equivalent ligand binding in both active sites of the dimer (Figs. 6 and 7*D*), which was directly comparable with the complex obtained by cocrystallization of *E. coli* AMY with acarbose (PDB code 4S3R) (29). In fact, each monomer closely resembled ACR-B, both in terms of the conformations of the gate, brace, and gatepost motifs and in the non-covalent binding of the ligand in the active site cleft. The only significant difference was in the conformation and positioning of the −4 and −3 sugars, which constituted an acarviosin moiety in ACV but had been built as maltose in ACR-B (due to the lack of convincing evidence to the contrary; Fig. 7, *C* and *D*). These structural variations in the ligands would be consistent with the identities of the −4 and −3 sugars being different between ACV-A and ACR-B (discussed further below).

##### AtDPE1 β-Cyclodextrin Soak

A further structure (BCD) was determined to 2.3 Å resolution after soaking an AtDPE1 crystal in β-cyclodextrin. In both active centers, there was only sufficient electron density to confidently build a maltose unit occupying subsites −2 and −1, this being covalently linked to Asp-373, and overlapping closely with the sugars in the equivalent subsites in the donor pocket of ACR-A (Fig. 7*E*). Weaker electron density was observed beyond and contiguous with that for the −2 sugar, indicative of a −3 sugar residue. In contrast to the situation in the ACR-A, the acceptor site is vacant (Fig. 6), the gate is disordered, and the brace adopts a conformation comparable with that seen in MA3 and LF (Fig. 5). The covalently bound donor most likely resulted from a slow hydrolysis of β-cyclodextrin because the soaking solution should have been devoid of linear glucans.

##### AtDPE1 Cycloamylose Soak

The remaining structure (CAM) was determined to 2.3 Å resolution after soaking an AtDPE1 crystal in cycloamylose. This was extremely similar to BCD-A, with a maltose unit occupying subsites −2 and −1, and covalently linked to Asp-373 in both active sites. Additionally, in CAM-B, the electron density clearly revealed a single glucose molecule occupying the +1 donor subsite (Fig. 7*F*), which overlapped well with the equivalent sugar in ACR-A (Fig. 7*B*). As with BCD-A, there was weak density extending beyond the −2 sugar in both subunits consistent with −3 sugar residues, whereas additional weak extended density was present in the acceptor site of CAM-B beyond the +1 sugar, suggesting the presence of +2 and +3 sugar residues, but these could not be modeled with confidence (Fig. 6). As with BCD-A, the gate is disordered, and the brace adopts a conformation comparable with that seen in MA3 and LF. In the cycloamylose sample used for crystal soaking, we cannot rule out the presence of contaminating linear α-glucans due to the noted sensitivity of cycloamylose to adventitious α-amylase; these would seem to be the most likely source of the bound ligands that we observe in this structure.

##### Transglycosylation of Acarbose

Previous work (20) has shownthat DPE1 catalyzes the transfer of the acarviosyl-glucose moiety from acarbose to suitable acceptors. If maltooligosaccharides are present, the resulting compounds are analogues of acarbose extended at its reducing end (20). We show that in the absence of maltooligosaccharide acceptors, the acarviosyl-glucose moiety can be transferred by AtDPE1 onto the non-reducing end of another acarbose, giving rise to the pseudo heptasaccharide acarviostatin II01 (see “Experimental Procedures” and Fig. 1*A*). After purification and characterization (Figs. 1*B* and 2), this compound was soaked into AtDPE1 crystals to yield the ACV-A structure. Although a similar soak with the starting material (*i.e.* acarbose (a pseudotetrasaccharide)) also showed density for a heptasaccharide in ACR-B, it was somewhat different from ACV-A at the non-reducing end (Fig. 7, *C* and *D*), suggesting that a different transglycosylation product had been trapped in ACR-B. However, given the resolution of the data for the latter (2.05 Å), we were unable to define the true identity of the bound ligand with confidence.

## Discussion

### Conformational Changes in AtDPE1

Through comparisons of the structures described herein, we are able to describe different conformations of AtDPE1, specifically for the gate and the brace motifs in the B1 subdomain and the gatepost in B3, and relate these to the occupancy of the various subsites in the active site canyon. In all cases, when the acceptor site is vacant, the end of the gate is disordered. In the structures where the acceptor site is occupied, the gate can adopt one of two discrete ordered conformations, depending on the catalytic state mimicked by the ligand complex (Fig. 8). The correlated movements of the gate and the brace that we observe essentially preserve a number of favorable contacts between these two motifs. Movement of the brace also propagates to the movement of the N-terminal arm of the opposing subunit through maintaining the integrity of the five-stranded β-sheet (Fig. 8*E*), potentially providing a mechanism whereby events at one active site are communicated to the other subunit. Therefore, it is tempting to speculate that this could explain the existence of distinctly different catalytic states between the two active sites within the same homodimer (*e.g.* ACR-A *versus* ACR-B). However, the changes at the N-terminal arm do not appear to be transmitted to the core of the subunit, and the relationship between protomers does not change significantly between the various AtDPE1 structures (Table 4). An alternative explanation for this asymmetry may involve the influence of crystal packing forces (*i.e.* the interactions between homodimers in the crystal). In particular, the brace motifs of each subunit lie close to crystal contacts such that, dependent on the structure and the subunit, they make different interactions with the neighboring dimer in the crystal. Thus, these crystal contacts could influence the rigidity and positioning of the brace, which in turn could affect the placement of the gate and therefore affect binding events at the active site. Although one could argue that the resultant conformations of AtDPE1 are crystallographic artifacts, we propose that these crystal contacts have helped us to trap conformations that mimic biologically relevant states of inherently dynamic structural motifs.

**TABLE 4:** **Comparison of AtDPE1 structures**

Model	Root mean square deviations		
	Dimer*^a^*	A subunit*^b^*	B subunit*^c^*
	Å	Å	Å
MA3	0.00	0.00	0.34
LF	0.51	0.46	0.37
ACR	1.01	0.72	1.13
ACV	1.20	1.10	1.05
BCD	0.27	0.27	0.35
CAM	0.35	0.34	0.41

*^a^* Values after superposition of dimer onto MA3 dimer.

*^b^* Values after superposition of A subunit onto MA3 A subunit.

*^c^* Values after superposition of B subunit onto MA3 A subunit.

### Configuration of Catalytic Residues

The observation that Asp-373 adopts a catalytically incompetent configuration upon substrate capture seen in MA3-A (Fig. 10*B*) is intriguing, but this is also seen in four of the homologous enzymes, suggesting that it may have biological relevance. In these homologues, either the active site cavity is vacant (*S. tuberosum* DPE1, *T. thermophilus* AMY, and *E. coli* AMY), or the substrate/product is bound in a pre/post-catalytic complex, analogous to MA3-A, where it is not fully engaged with the active site (*T. aquaticus* AMY, *E. coli* AMY maltose complex). Notably, in all of these structures, the carboxylate of the Asp-373 equivalent is within hydrogen bonding distance of another conserved Asp side chain (Asp-293 in AtDPE1). This implies that one of these acids is protonated and therefore has an elevated p*K_a_*, which is particularly remarkable given that the *T. aquaticus* AMY crystals were grown at pH 9.0 (25). Indeed, in both MA3-A and *T. thermophilus* AMY, the interatomic distance is 2.4 Å, and it is only 2.25 Å in *T. aquaticus* AMY, suggestive of low barrier hydrogen bonds where the p*K_a_* values of the participants are matched, and they share the proton equally (54). The functional significance of this interaction, if any, is unclear at present. Moreover, in all of the amylomaltase structures, except the acarbose complex of *E. coli* AMY (PDB code 4S3R) (29), and all but one of the AtDPE1 structures, although less striking, the carboxylate moiety of the acid/base catalyst (Glu-420 in AtDPE1) is also held in a catalytically incompetent configuration through hydrogen bonding to backbone nitrogen atoms of the protein segment containing itself (specifically with Leu-422 in AtDPE1) and that containing the nucleophile (specifically His-374 in AtDPE1), respectively (Fig. 10, *A*, *B*, and *D*). The exception is ACR-B, where O^ϵ1^ retains the hydrogen bond to His-374 but swivels round so that O^ϵ2^ can now hydrogen-bond to the nitrogen of the acarviosin moiety of acarbose, thereby mimicking the expected interaction with the oxygen of the scissile glycosidic bond, which would be a prerequisite to catalysis (Fig. 10*C*; the equivalent changes are seen for Glu-496 in the acarbose complex of *E. coli* AMY). This side chain movement could be triggered by a small shift in the protein backbone downstream of Glu-420 (highlighted in Fig. 5), which may result in the loss of the hydrogen bond between O^ϵ2^ and the backbone nitrogen of Leu-422, thereby removing a constraint on the side chain. Moreover, in ACR-B, Asp-373 is now optimally poised for nucleophilic attack on the anomeric carbon, lying only 2.8 Å from it (Fig. 10*C*). The reconfiguration of the active site acids into catalytically competent conformations is most likely driven by a series of conformational changes that are coupled to the proximity of an appropriately bound substrate, but we are not able to precisely define the necessary sequence of events.

### Mechanistic Implications of AtDPE1 Structures

**FIGURE 11.**
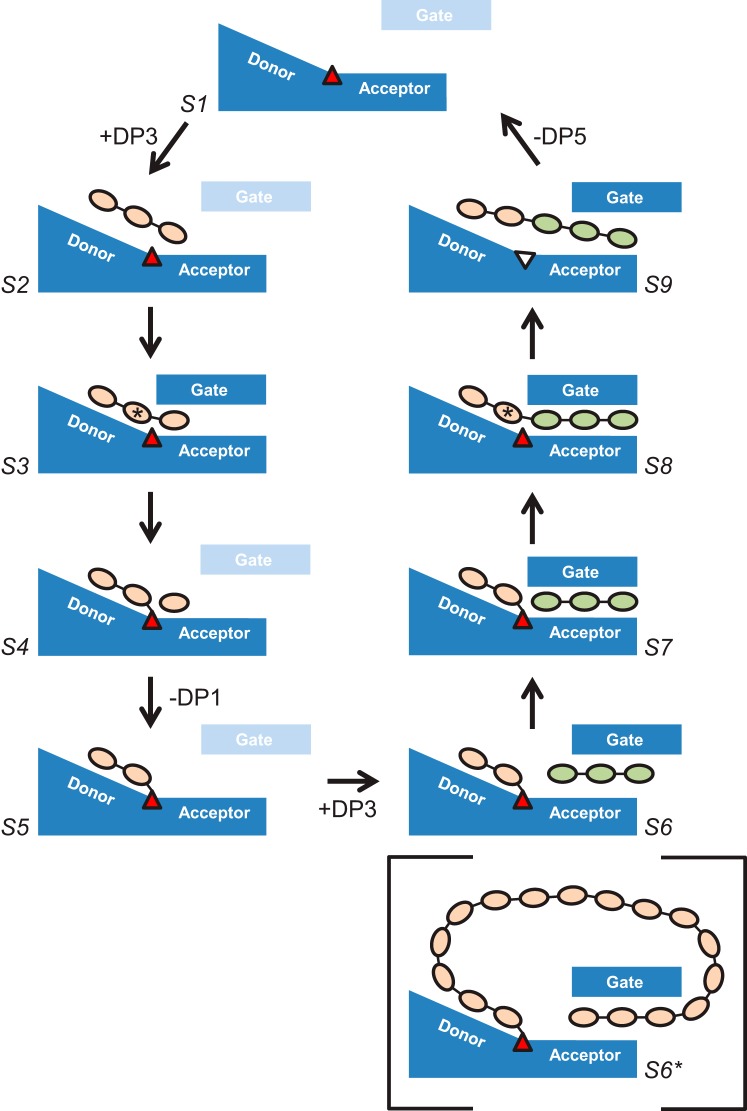
**A putative scheme for AtDPE1-catalyzed transglycosylation.** Shown is a schematic representation of the various states (S1–S9) proposed for the disproportionation of two molecules of maltotriose by AtDPE1 to yield glucose and maltopentaose. The gate is shown either as disordered (*pale blue*) or in a partially or a fully closed conformation and may play a more significant role in the second half-reaction. Also shown (S6*) is a putative intermediate state, where the gate is involved in capturing the non-reducing end of a long donor molecule in the acceptor site to yield a cyclic product. All sugars are expected to adopt a ^4^C_1_ chair conformation except at the −1 subsites in the two transition states where a half-chair conformation is expected (indicated by the *asterisks*). The *red triangle* indicates the active site nucleophile (Asp-373), which is shown in an incompetent conformation (and *colored white*) in the post-catalytic state S9, in accordance with the MA3-A structure upon which this is based (see Figs. 9*A* and 10*B*). By contrast, we speculate that in the precatalytic substrate-bound state S2, the nucleophile adopts a catalytically competent conformation.

For an enzyme that has evolved to transglycosylate, the intermediate state needs to be relatively long lived to allow for exchange of the leaving group and the acceptor molecule. This would be consistent with the relatively stable ^4^C_1_ chair adopted by the −1 sugar of the covalently bound donor moiety (Fig. 10*D*) (55). However, because the intermediate would be vulnerable to abortive hydrolysis, it seems plausible that the enzyme has developed strategies to make this less likely. The first of these may be to adopt a catalytically incompetent configuration for the acid/base catalyst (*i.e.* Glu-420) in the absence of a suitable acceptor, as we observe in all but the ACR-B structure, and thereby preventing it from activating a water molecule and leading to hydrolysis. A second protective mechanism could result from the perpendicular arrangement of the plane of the nucleophilic carboxylate group to the plane of the −1 sugar, such that the anomeric carbon is partially occluded and less accessible to an approaching nucleophile, as noted in *T. thermophilus* AMY (27), which we observe in ACR-A, BCD-A, and CAM-B (Figs. 7 (*B*, *E*, and *F*) and 10*D*). It is also possible that the gate may play a part in protecting the intermediate from abortive hydrolysis. Indeed, the fact that we are able to trap the covalently bound intermediates at all is testament to their stability. This contrasts with the situation in related enzymes, where it is usually necessary to make a mutation to the acid/base catalyst and to use fluorinated pseudosubstrates in order to trap a covalent intermediate (56–58). In general, the incompetent configurations of both the nucleophile and acid/base catalyst may help to avoid undesirable reactions, such as re-reacting with products that have not left the active site or reacting with longer glucan chains such that the leaving group is larger than maltose, and therefore not a transporter substrate, and the product is shorter than the substrate. Indeed, this may be the reason for the incompetent configuration of Asp-373 seen in MA3-A, where we see maltononaose bound, and could explain the loss of DPE1 activity observed with progressively longer glucan chains as substrates (10). Perhaps the partially closed gate is the trigger for the incompetent conformation of the nucleophile in such complexes. By contrast when the preferred substrate (*i.e.* maltotriose) binds in the correct register, the catalytically competent active site configuration is desirable, as we have predicted for state S2 in our proposed scheme below (Fig. 11).

### A Putative Scheme for AtDPE1-catalyzed Transglycosylation

The expected α-retaining double-displacement mechanism of AtDPE1 consists of two half-reactions, with a covalently bound β-linked intermediate lying at the midpoint of the scheme. Observations that AtDPE1 incubated with maltotriose never produces maltose (9) and that maltose cannot serve as a substrate (10) are consistent with binding to subsite −2 as well as to subsites −1 and +1 being necessary for catalysis to occur. In addition, all currently available structural data indicate that when subsite −1 is occupied, so too is subsite −2 (Figs. 6 and 7). Therefore, with maltotriose as substrate, this would be expected to span subsites −2 to +1 and go on to liberate glucose with the concomitant production of a maltosyl-enzyme intermediate. In the second half-reaction, a further maltotriose molecule would then act as the acceptor, occupying subsites +1 to +3, to generate maltopentaose upon transglycosylation. It is clear from our ligand-bound structures that the most extensive interactions with the protein are made with sugars occupying the −2, −1, and +1 subsites (Fig. 9), this being consistent with a preference for maltotriose as substrate. Furthermore, our structural data show that the gate motif is flexible, and, in the absence of sugar residues occupying the acceptor pocket beyond subsite +1, it is disordered. Therefore, in the case where maltotriose is the substrate, we predict that the gate plays a lesser role in the first half-reaction. Instead, it seems likely that the gate assists in the capture of the acceptor maltotriose molecule and in correctly aligning it with the covalently bound maltosyl unit in preparation for transglycosylation. Based on these assumptions and guided by our x-ray structures, we propose the following scheme for the disproportionation of two molecules of maltotriose into maltopentaose and glucose by AtDPE1, as outlined in Fig. 11.

#### 

##### S1. Free Enzyme; Disordered Gate

This is the status of the enzyme prior to reaction and represented by the LF-A structure.

##### S2. Precatalytic State; Maltotriose Bound; Disordered Gate

In this state, the substrate is not fully engaged with the active site. This could resemble MA3-A but with just the sugar residues spanning subsites −2 to +1, making multiple favorable interactions with the protein (Fig. 9*A*). Moreover, because sites +3 and +4 are vacant, the gate is likely to be disordered. In addition, because this is the preferred substrate, we propose that Asp-373 is correctly configured for catalysis (in contrast to MA3-A).

##### S3. Transition State of the First Half-reaction; Maltotriose Fully Engaged with the Active Site (−1 Sugar Adopts a Half-chair Conformation); Fully Closed Gate

This could resemble ACR-B (and ACV-A) but with just the sugar residues spanning subsites −2 to +1. The flipping of the −1 sugar from a ^4^C_1_ chair to a transition state-like half-chair is stabilized by the interactions with the protein, in particular the bidentate interaction between Asp-473 and O2 and O3. This introduces a kink into the glucan chain at the scissile linkage that allows the substrate to be drawn deeper into the active site. At the same time, the flattening of the −1 ring and its partially aromatic character attracts the side chain of Trp-338 down with it. In addition, the deeper repositioning of the +1 sugar pulls the side chain of Gln-336 down too. Both Gln-336 and Trp-338 lie at the base of the gate, and their combined shifts could act as a trigger that initiates the movement of the gate from a disordered to a fully closed configuration. Additionally, the repositioning of the gatepost is necessary for the optimal stacking of Phe-331 against Pro-539, which may be aided through the correlated motion of the adjacent residue, Ala-540, with the substrate, such that its backbone nitrogen remains hydrogen-bonded to O3 of the −2 sugar as it moves downward in the donor site (Fig. 9*B*).

##### S4. Maltosyl-Enzyme Intermediate; Glucose in Subsite +1; Disordered Gate

This is represented by CAM-B. The −1 sugar flips back to a ^4^C_1_ chair upon forming the β-glycosidic link with Asp-373, so the interaction with Trp-338 is weakened, and the gate would become more likely to open, especially since the +1 sugar is less constrained due to the loss of the glycosidic bond.

##### S5. Maltosyl-Enzyme Intermediate; Vacant Acceptor Site; Disordered Gate

This is represented by BCD-A. The release of glucose primes the enzyme to receive the second maltotriose molecule.

##### S6. Maltosyl-Enzyme Intermediate; Second Maltotriose Partially Engaged with the Acceptor Site; Partially Closed Gate

The donor site could be represented by BCD-A, and the acceptor site could be represented by MA3-A, specifically from the latter, the sugar residues spanning subsites +1 to +3 with the partially closed gate trapping the +3 sugar between Phe-331 and Phe-446.

##### S7. Maltosyl-Enzyme Intermediate; Second Maltotriose Fully Engaged with the Acceptor Site; Fully Closed Gate

This is represented by ACR-A but with just the sugar residues spanning subsites −2 to +3. The fully engaged acceptor molecule, in addition to drawing down Gln-336 to trigger full gate closure, makes further favorable contacts with Asp-329 and Leu-330 of the repositioned gate via the sugar residues in subsites +2 and +3 (Fig. 9*C*).

##### S8. Transition State of the Second Half-reaction; Maltopentaose Fully Engaged with the Active Site (−1 Sugar Adopts a Half-chair Conformation); Fully Closed Gate

This is represented by ACR-B (and ACV-A) but with just the sugar residues spanning subsites −2 to +3. The β-link with Asp-373 at the reducing end of the donor maltose is replaced by an α-1,4-link to the non-reducing end of the acceptor maltotriose molecule to yield maltopentaose, and the −1 sugar adopts a half-chair conformation. The favorable contacts between Gln-336, Asp-329, and Leu-330 and the acceptor site sugars maintain the fully closed gate conformation (Fig. 9*B*).

##### S9. Post-catalytic State; Maltopentaose Partially Engaged with the Active Site; Partially Closed Gate

This is represented by MA3-A but with just the sugar residues spanning subsites −2 to +3. The reversion of the −1 sugar to a ^4^C_1_ chair conformation removes the kink at the newly formed glycosidic linkage such that the maltopentaose can no longer fully engage with the active site. The outward movement of the product and the concerted movements of residues around the −1 and +1 sites (*i.e.* Ala-540, Gln-336, and Trp-338) cause the gate and gatepost to move apart, with the +3 sugar residue possibly being transiently clamped between Phe-331 in the partially closed gate and Phe-446 as we see in MA3-A (Figs. 8*A* and 9*A*).

##### S1. Free Enzyme; Disordered Gate

The opened gate allows the maltopentaose to disengage to yield the free enzyme ready for another catalytic cycle.

### The Production and Significance of Cycloamyloses

To date, there is no direct evidence for the production of cyclic products by DPE1 in plants. Nevertheless, *in vitro*, upon prolonged incubation with maltotriose, repeated transglycosylation is possible to yield longer and longer glucan chains and, eventually, cyclic products (14). Given that both the donor and acceptor sites are open-ended in AtDPE1, a mechanism whereby cyclization could be achieved is entirely conceivable (Fig. 11, state S6*). We have previously shown that AtDPE1 is capable of producing cycloamyloses ranging from 16 to 50 glucose units in size, with DP 18 products being the most abundant (11). Similarly, products of DP 17 up to several hundred have been recorded for the potato tuber enzyme (14). An analogous scheme to that proposed for intermolecular transglycosylation above is applicable to the intramolecular transglycosylation necessary to result in cyclization. Steps S2–S5 would essentially be the same, except that the donor portion would be much longer. In order to give a DP 18 cycloamylose, a DP 19 substrate would be required, assuming that only the +1 acceptor site is occupied prior to forming the intermediate (for greater overlap with the acceptor pocket, correspondingly larger substrates would be required to yield the same cyclic product). We assume that the majority of the non-reducing end would be highly mobile, enabling it to wrap around and be captured by the gate, as shown in step S6* (Fig. 11). Thereafter, cyclization would be achieved through steps analogous to S7–S9, except in this case the “acceptor” is actually the non-reducing end of the donor. In this revised scheme, the gate would be expected to play the same roles in acceptor capture and positioning. Indirectly, it would also have the effect of limiting the minimum size of cycloamylose produced due to the requirement to wrap around the gate. Indeed, it has already been proposed that the gate may prevent the formation of small cyclic products in amylomaltases, and, based on the structure of *T. aquaticus* AMY (PDB code 1CWY) (26), it was estimated that if the cycloamylose product wraps around the gate, the smallest ring size might be DP ∼18. By contrast, in cyclodextrin glycosyltransferases, there is no equivalent of the gate, and thus smaller cyclic products in the range DP 6–8 are possible. Given that AtDPE1 can readily form cycloamyloses from maltotriose *in vitro* and that the MA3 structure was determined from a crystal soaked for several hours in the latter, it is entirely possible that what we see in the active sites of this structure is simply the crystallographically resolved portions of much larger cycloamyloses.

#### 

##### Conclusion

The set of crystal structures presented herein allows us to delineate the complete catalytic cycle of AtDPE1 in structural terms. Notably, we demonstrate the capture of covalent intermediates without resorting to site-directed mutants or exotic substrate analogues. We describe the orchestrated movements of surface loops and catalytic residues that together direct the disproportionation of short linear glucans, and we propose a mechanism whereby longer substrates could be cyclized. Although no biological role for cycloamyloses has so far been described, they have a range of biotechnological applications. Moreover, besides cyclization, this class of enzyme is capable of performing a variety of other industrially relevant biotransformations *in vitro* via transglycosylation. Thus, our results will inform the future practical use of AtDPE1 or its homologues in a range of green chemistry applications.

## Author Contributions

E. C. O., K. T., and C. E. M. S. produced, characterized, and crystallized the protein; E. C. O., C. E. M. S., and D. M. L. collected and analyzed the x-ray data; D. L., M. I. D., M. R., and S. A. N. produced and characterized the acarviostatin; T. L. and R. A. F. conceived and supervised the project; D. M. L. drafted the manuscript with assistance from E. C. O.; all authors contributed to editing the manuscript.

## Footnotes

5 K. Imamura, T. Matsuura, T. Takaha, K. Fujii, A. Nakagawa, M. Kusunoki, and Y. Nitta, unpublished results.

6 T. R. M. Barends, H. Korf, T. Kaper, M. J. E. C. van der Maarel, L. Dijkhuizen, and B. W. Dijkstra, unpublished results.

7 L. Wu, J. Zhou, J. Wu, J. Li, and J. Chen, unpublished results.

## Footnotes

4 The abbreviations used are:

DPE1: disproportionating enzyme 1
ACR: AtDPE1 structure from acarbose soak
ACV: AtDPE1 structure from acarviostatin soak
AtDPE1: *A. thaliana* disproportionating enzyme 1
BCD: AtDPE1 structure from β-cyclodextrin soak
CAM: AtDPE1 structure from cycloamylose soak
DP: degree of polymerization
LF: ligand-free structure of AtDPE1
MA3: AtDPE1 structure from maltotriose soak
MA3-A: A subunit from MA3 structure
MA3-B: B subunit from MA3 structure
PDB: Protein Data Bank
AMY: amylomaltase.
